# Insulin is central, plasmapheresis is salvage: clinical spectrum and management of severe hypertriglyceridemia in 21 children with diabetic ketoacidosis

**DOI:** 10.3389/fendo.2026.1920206

**Published:** 2026-08-26

**Authors:** Meijuan Liu, Di Wu, Chang Su

**Affiliations:** Department of Endocrinology, Genetics and Metabolism, Beijing Children’s Hospital, Capital Medical University, National Center for Children’s Health, Beijing, China

**Keywords:** acute pancreatitis, children, diabetic ketoacidosis, insulin, plasmapheresis, severe hyperlipidemia

## Abstract

**Background:**

Severe hypertriglyceridemia (SHTG), characterized by plasma triglycerides (TG)>11.3 mmol/L is a rare but serious complication in children who develop diabetic ketoacidosis (DKA). However, the current body of evidence is predominantly composed of case reports, highlighting a substantial gap in both the understanding and management of this condition.

**Methods:**

This was a retrospective electronic medical record review of pediatric patients with DKA complicated by SHTG who were referred to the Beijing Children’s Hospital over a 10-year period.

**Results:**

A total of 21 pediatric patients with DKA complicated by SHTG were identified, with a male-to-female ratio of 8:13. The median age at diagnosis was 11.3 years [interquartile range (IQR) 10.5-13.2, range 2.0-17.4], with approximately 76.2% (16/21) of patients aged ≥10 years. Among these patients, 10 were diagnosed with type 1 diabetes mellitus, 5 with type 2 diabetes mellitus, and 6 with unclassified diabetes mellitus. 17 patients were newly diagnosed with diabetes, whereas 4 had a history of pre-existing diabetes. Notably, 3 of these 4 patients had discontinued insulin prior to admission. In terms of complications, 7 patients had acute pancreatitis. Regarding treatment, all patients received insulin therapy, among whom 7 patients were treated with plasmapheresis. Compared with insulin therapy alone, the plasmapheresis group showed a shorter median time to achieve the TG level <5.6 mmol/L, though this finding should be interpreted cautiously due to potential selection bias. Among the 7 patients who received plasmapheresis, 4 developed venous thrombosis. During follow-up, patients who underwent plasmapheresis maintained stable lipid profiles and achieved complete resolution of thrombosis.

**Conclusion:**

We present the largest single-center experience on SHTG in pediatric patients with DKA, with a particular focus on plasmapheresis. We recommend early assessment of dyslipidemia and pancreatic enzymes in patients with DKA. Insulin therapy remains the cornerstone of management. However, in children who show unsatisfactory reduction in triglyceride levels or persistent abdominal pain despite insulin treatment, prompt initiation of plasma exchange may be considered as a rescue therapy to rapidly lower TG levels, which could potentially mitigate the risk of developing or worsening acute pancreatitis. This hypothesis requires validation in larger prospective studies.

## Introduction

Epidemiological data from both global and Chinese studies demonstrate a rapidly increasing incidence of diabetes mellitus (DM) among children (1–3). Diabetic ketoacidosis (DKA), a critical and potentially life-threatening acute complication, often arises from insulin deficiency in cases of new-onset or poorly controlled diabetes. This deficiency triggers a cascade of counter-regulatory hormone responses, resulting in hyperglycemia, ketosis, and acidosis (4). Beyond its classic metabolic disturbances, DKA can precipitate severe complications such as cerebral edema, stroke, and hypovolemic shock (5).

Lipid abnormalities, specifically severe hypertriglyceridemia [SHTG; triglyceride (TG) >11.3 mmol/L], are another potential complication of patients with DKA due to insulin deficiency (6). Although the pathophysiological mechanisms linking insulin deficiency to impaired lipoprotein lipase activity and increased lipolysis are well established (7), the clinical implications of SHTG in pediatric DKA remain inadequately characterized. In adults, the prevalence of SHTG in conjunction with DKA has been reported to be approximately 11-12.5% (8, 9), with the risk of acute pancreatitis in this context estimated at 5% (10). Nevertheless, comprehensive epidemiological data concerning the pediatric population remain limited. A prospective study indicated that hypertriglyceridemia (>2.26 mmol/L) may occur in up to 40% of children with DKA (11). However, SHTG is exceedingly rare, with only isolated case series published to date (12, 13).

Current management strategies for SHTG in pediatric DKA remain controversial. The primary therapeutic approach for both DKA and concomitant SHTG involves the prompt administration of insulin and fluid resuscitation, which addresses the underlying insulin deficiency (14). However, previous pediatric case studies concerning this clinical triad have documented an extended duration for the reduction of TG levels when relying solely on intravenous fluids and insulin therapy, with some patients requiring more than 15 days to achieve target levels (14, 15). In selected pediatric cases, particularly those with concurrent pancreatitis, therapeutic plasma exchange has been employed to rapidly lower TG levels (13, 16–18). To date, only 13 pediatric cases of DKA complicated by SHTG and pancreatitis have been reported in the literature, with plasma exchange successfully used in a minority of these cases (17). The precise role of plasma exchange in preventing pancreatitis or the improvement of outcomes in children without overt pancreatitis remains highly uncertain, and there is a lack of definitive pediatric guidelines to guide clinical decision-making regarding its application. The optimal timing, indications, and risk-benefit profile of plasma exchange in this population are yet to be defined.

To address this gap in knowledge, we reviewed the clinical management and outcomes of 21 children with DKA complicated by SHTG, with a particular focus on 7 cases who underwent plasma exchange. To our knowledge, this represents the largest single-center pediatric series reported to date. Our findings aim to clarify the clinical spectrum of this complication and contribute to a more evidence-based approach for managing SHTG in pediatric DKA. We emphasize the importance of early, aggressive treatment with insulin, while also delineating the potential role of plasma exchange as a rescue therapy.

## Methods

### Subjects

**Figure 1:**
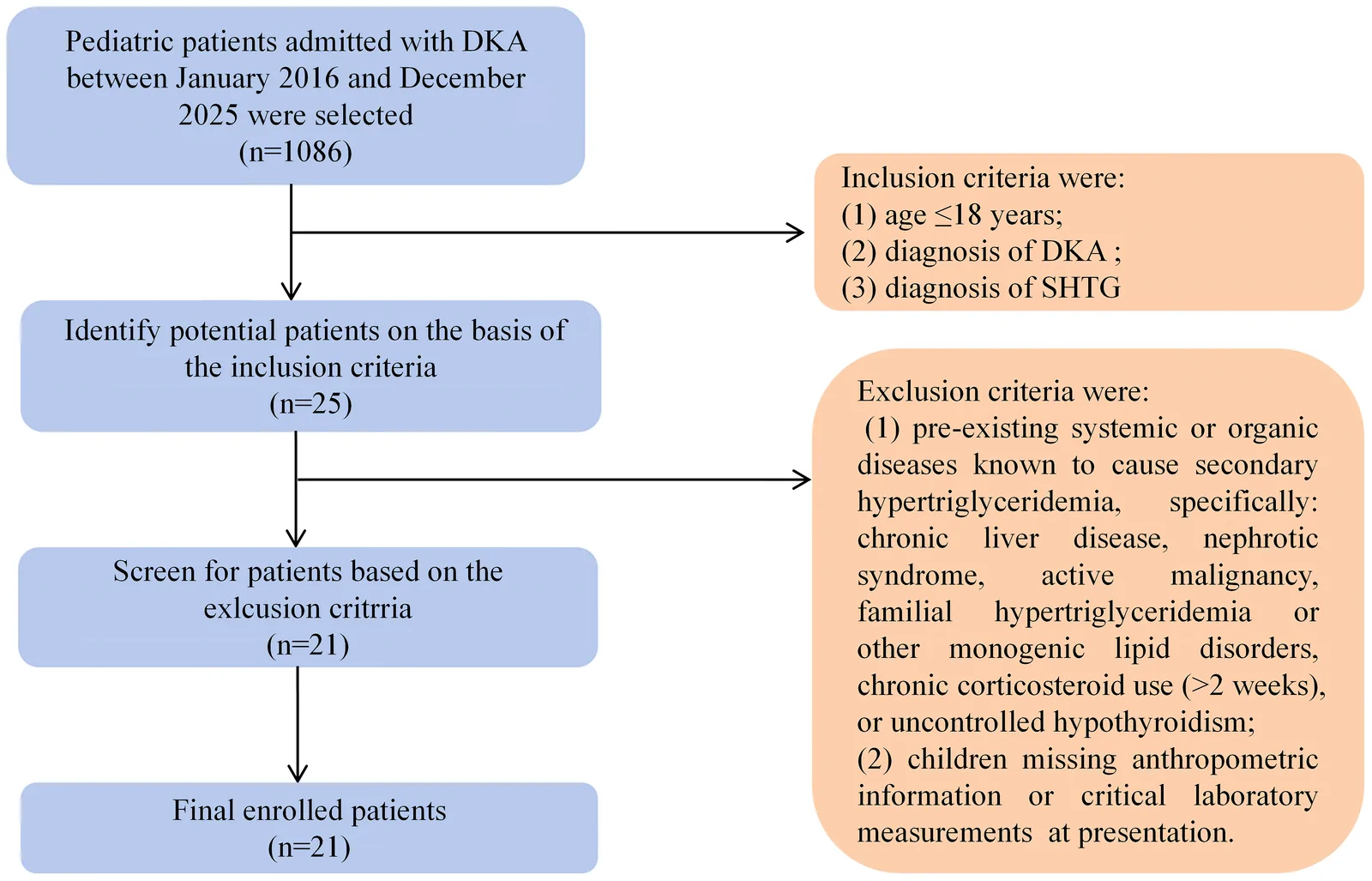
Flow chart for patient section.

This was a retrospective observational cohort study conducted at a single tertiary care center (Beijing Children’s Hospital, Capital Medical University). We systematically reviewed the electronic medical records of all pediatric patients admitted with DKA between January 2016 and December 2025. During the study period, a total of 1086 DKA admissions were identified. Routine assessment of TG levels was conducted for all patients. TG concentrations were determined using blood samples collected upon arrival at the emergency department under non-fasting conditions, consistent with the standard clinical practice for patients presenting with DKA. Flow chart for patient selection is shown in **Figure 1**. Inclusion criteria were: (1) patients aged ≤18 years; (2) diagnosis of DKA based on the 2022 International Society for Pediatric and Adolescent Diabetes (ISPAD) criteria, which include hyperglycemia (blood glucose >11 mmol/L), metabolic acidosis (venous pH <7.3 or serum bicarbonate <15 mmol/L), and presence of ketones in blood or urine (4); (3) SHTG was defined as a TG level exceeding 11.3 mmol/L in accordance with the guidelines of the Endocrine Society (19). Exclusion criteria were as follows: (1) pre-existing systemic or organic conditions known to induce secondary hypertriglyceridemia, specifically: chronic liver disease (e.g., cirrhosis, hepatitis), nephrotic syndrome, active malignancy, familial hypertriglyceridemia or other monogenic lipid disorders, chronic corticosteroid use exceeding 2 weeks, or uncontrolled hypothyroidism; (2) children missing anthropometric data or essential laboratory measurements, such as TG, lipase, amylase at the time of presentation. Ultimately, a total of 21 children (8 boys and 13 girls) were enrolled in this study. Acute pancreatitis was diagnosed based on the presence of at least two of the following three criteria, consistent with the revised Atlanta classification adapted for pediatrics (20, 21): (1) abdominal pain indicative of pancreatitis, characterized by an acute onset of epigastric pain often radiating to the back, with or without nausea or vomiting; (2) serum lipase and/or amylase activity elevated to at least three times the upper limit of normal; (3) imaging findings consistent with acute pancreatitis observed through abdominal ultrasound, computed tomography, or magnetic resonance imaging, which may include findings such as pancreatic enlargement, peripancreatic fat stranding, necrosis, or fluid collections.

### Plasmapheresis

The decision to initiate therapeutic plasma exchange was collaboratively made by the attending pediatric endocrinologist and a pediatric intensivist. Indications included any of the following: (1) persistent or worsening abdominal pain despite intravenous insulin therapy; (2) TG levels >22.6 mmol/L with no significant decline (<20%) after 24 hours of insulin administration; (3) evidence of acute pancreatitis irrespective of TG levels; (4) multi-organ dysfunction (e.g., acute kidney injury, respiratory failure, shock) attributed to hypertriglyceridemia. Plasma exchange was performed using a continuous flow centrifugal system (Spectra Optia, Terumo BCT). Vascular access was established via a temporary central venous catheter, either in the internal jugular or femoral vein. Fresh frozen plasma was used as the replacement fluid during the initial session, switching to 5% albumin for subsequent sessions if coagulopathy was absent. Anticoagulation was maintained using citrate at a whole-blood to anticoagulant ratio of 12:1. Each session processed 1.0–1.5 times the estimated plasma volume. The number of sessions ranged from 1 to 2, determined by achieving a post-exchange TG level <11.3 mmol/L and the resolution of clinical symptoms.

### Insulin therapy

All patients were administered intravenous regular insulin at an initial infusion rate of 0.1units/kg/hour, which was adjusted within the range of 0.05–0.10 units/kg/hour to maintain a glucose decline of 2.8–5.6 mmol/L/hour. Upon resolution of DKA, defined as a venous pH >7.3, serum bicarbonate >15 mmol/L, and negative urine ketones on two consecutive tests, the insulin infusion was continued at the same or a slightly reduced rate until TG levels decreased below 5.6 mmol/L. Subsequently, patients were transitioned to subcutaneous insulin using a basal-bolus regimen, with an overlap period of 1–2 hours. Fluid resuscitation was conducted in accordance with the ISPAD 2022 guidelines, beginning with an initial administration of 10–20 mL/kg isotonic saline, followed by deficit replacement over a 48-hour period (4). No lipid-lowering medications were used during the acute phase.

### Anthropometric measurements and laboratory examinations

Sex, age at diagnosis, body height, weight, body mass index (BMI), blood gas, biochemical analysis, β-hydroxybutyric acid and pancreatic enzymes on admission, HbA1c, presence of diabetes-associated autoantibodies [islet cell antibody (ICA), glutamic acid decarboxylase (GAD), insulinoma antigen-2 (IA-2), and insulin autoantibody (IAA)], type of diabetes, and treatment were recorded. The BMI Z-score was calculated based on the United States Centers for Disease Control and Prevention (CDC) growth reference. TG levels were monitored every 1–2 days until they decreased below 5.6 mmol/L, after which measurements were conducted weekly. Lipase and amylase concentrations were measured using standardized enzymatic assays, while β-hydroxybutyrate levels were quantified using an enzymatic assay.

### Statistical analyses

Descriptive statistics were applied to characterize baseline demographic, clinical, and biochemical profiles of the cohort. The Shapiro-Wilk test was used to test the normality of distribution for continuous variables. Variables with skewed distribution were expressed as median and interquartile range (IQR). The Mann-Whitney U test was utilized to evaluate differences between groups. Categorical variables were summarized as frequencies and percentages [n (%)], with comparisons between groups performed using Fisher’s exact test. Missing data were present for a small number of laboratory variables (indicated as ‘NA’ in **Table 1**). All analyses were performed using pairwise deletion; that is, each comparison included only patients with available data for the specific variable. Given the limited sample size (n=21) and the small number of patients receiving plasmapheresis (n=7), multivariable regression analyses were not performed, as such models might yield unreliable estimates rather than meaningful adjustment. All statistical procedures were completed using IBM SPSS Statistics version 26.0 (IBM Corp., Armonk, NY, USA). A two-sided *P*-value <0.05 was defined as statistically significant.

**Table 1:** Biochemical characteristics of patients with DKA complicated by SHTG.

Patients	PH	HCO3- (mmol/L)	β-hydroxybutyric acid	FBG (mmol/L)	Cr (umol/L)	TG (mmol/L)	TC (mmol/L)	HDL-C (mmol/L)	LDL-C (mmol/L)	AST (U/L)	ALT (U/L)	Amylase (IU/L)	Lipase (U/L)	HbA1c(%)	Antibody
1	7.3	14.8	6.9	26.2	18.4	26.8	10.8	1.0	3.2	52.2	50.2	50	51.9	15.1	IA-2
2	7.3	18.4	4.4	20.1	67.2	15.7	5.5	0.7	2.0	35.4	87.1	63	176.6	10.1	–
3	7.3	9.6	2.46	14.4	36.6	30.3	11.0	0.7	4.8	44.8	12.1	354	1006.7	15	–
4	6.9	4.9	5.7	6.7	53.6	15.7	11.3	0.9	3.7	35.3	6.7	117	NA	16.4	GAD
5	7.3	13.2	1.2	9.5	27.3	20.3	10.8	1.0	4.3	15.1	16.5	125	198.6	10.9	–
6	7.1	7.5	0.8	11.5	167.5	21.6	17.7	0.9	6.4	49.9	24.7	34.0	90.3	18.9	–
7	7.3	11.3	2.2	13.5	33.5	17.8	8.0	1.8	2.7	21.5	22.5	175.6	79	13.2	–
8	7.2	7.8	8.2	20.1	35.8	13.3	7.7	0.9	3.0	17.0	11.3	NA	NA	13.4	GAD
9	7.1	4.8	4.4	22.0	48.7	24.2	14.0	0.9	3.7	18.5	24.4	26.0	40.1	15.6	–
10	7.2	12.7	10.3	75.8	25.8	33.0	13.5	1.0	3.1	12.8	13.3	47.0	82.2	15.5	–
11	7.1	4.6	10.4	34.5	42.0	11.3	7.5	1.7	2.4	18.5	18.4	14.6	43.0	15.5	–
12	7.3	11.4	7.7	35.0	14.1	15.6	7.8	2.0	2.9	27.3	26.6	NA	NA	13.5	–
13	6.8	3	10.4	28.8	55.0	11.1	6.3	1.3	2.6	11.8	10.2	724.5	234.0	11.3	–
14	7.2	8.2	9.5	14.4	21.2	16.2	8.5	2.5	2.6	21.8	23.0	NA	NA	14.8	–
15	7.0	6.4	11.0	29.5	54.2	16.5	10.8	0.8	4.1	49.6	18.7	486	254.5	14.9	GAD
16	6.9	3.9	9.4	21.4	56.8	53.0	14.8	1.0	6.0	55.2	16.8	81	87	13.3	IA-2
17	7.3	13.2	NA	11.6	36.9	30.9	13.1	0.6	5.5	30.0	20.2	146	137.1	17.1	GAD
18	7.1	4.8	9.2	12.0	54.6	41.3	13.4	0.7	3.9	39.2	18.4	17	140.7	10.8	–
19	7.2	12.0	5.0	15.6	28.5	39.3	16.2	0.8	6.6	40.2	43.9	227.0	656.3	12.4	–
20	7.1	12.1	4.1	15.9	65.8	69.3	15.8	0.6	5.6	19.2	6.7	150.0	465.5	13.4	–
21	6.7	3.9	NA	25.4	51.3	35.0	12.0	0.8	3.1	28.2	22.4	47.0	52.2	10.6	–

DKA, diabetic ketoacidosis; SHTG, severe hypertriglyceridemia; FBG, fasting blood glucose; Cr, creatinine; TG, triglycerides; TC, total cholesterol; HDL-C, high density lipoprotein cholesterol; LDL-C, low density lipoprotein cholesterol; AST, aspartate aminotransferase; ALT, alanine aminotransferase; IA-2, insulinoma antigen-2; GAD, glutamic acid decarboxylase; NA, not available.

## Results

21 pediatric cases with DKA complicated by SHTG were identified in our center. The median age at diagnosis of 21 cases was 11.3 years (IQR 10.5-13.2, range 2.0-17.4). Of these patients, 62.0% (n=13) were female, and 76.2% (n=16) were older than 10 years. The cohort included 10 patients diagnosed with type 1 diabetes mellitus (T1DM, 47.6%), 5 patients with type 2 diabetes mellitus (T2DM, 23.8%), and 6 patients with unspecified DM (28.6%) (**Supplementary Table 1**). Among the 21 patients, 17 were newly diagnosed with diabetes, whereas 4 had a history of pre-existing diabetes. Notably, 3 of these 4 patients had self-discontinued insulin therapy prior to this admission. The median peak TG level was 21.6 mmol/L (IQR 15.7-33.0 mmol/L, range 11.1-69.3 mmol/L). Liver function injury, indicated by alanine aminotransferase (ALT) exceeding 40 IU/L, was observed in only 3 patients. Diabetes autoantibodies were detected in six patients (28.6%), with 4 testing positive for GAD antibody and 2 for IA-2 antibody (**Table 1**).

At the time of diagnosis, acute pancreatitis was documented in 7 patients (33.3%). The clinical characteristics of patients with DKA complicated by SHTG, with or without pancreatitis, were compared in **Table 2**. In comparison to individuals without pancreatitis, patients with pancreatitis had a higher median BMI Z-score (median 1.8, IQR 1.1–2.0 vs. median −1.2, IQR −1.8 to -0.1; median difference 3.0, 95% CI 1.4 -3.7, *P*<0.01) and higher lipase levels (median 198.6, IQR 158.6–560.9 vs. median 84.6, IQR 52.0–125.4; median difference 114.0, 95% CI 14.4-604.1, *P*=0.03). Conversely, patients with pancreatitis showed reduced levels of β-hydroxybutyric acid levels (median 4.1, IQR 2.3–4.7 vs. median 8.8, IQR 6.6–10.3; median difference −4.7, 95% CI −7.8 to −1.9, *P*=0.02) and lower HbA1c (median 12.4%, IQR 10.9–13.3 vs. median 15.0%, IQR 13.4–15.6; median difference −2.6, 95% CI −4.7 to −0.2, *P*=0.02) compared to those without pancreatitis. All 7 patients with pancreatitis were negative for diabetes autoantibodies (GAD, ICA, IAA, IA-2).

**Table 2:** Comparison of biochemical characteristics between DKA patients complicated by SHTG with or without pancreatitis.

Variables	Overall (n=21)	With acute pancreatitis (n=7)	Without acute pancreatitis (n=14)	Median difference (95% CI)	*P*-value
Age	11.3 (10.5-13.2)	12.4 (10.8-14.2)	11.2 (6.8-12.8)	1.2 (-1.8-6.6)	0.35
Male (n, %)	8 (38.1%)	4 (57.1%)	4 (28.6%)	/	1.00
BMI Z-score	-0.5 (-1.4-1.2)	1.8 (1.1-2.0)	-1.2 (-1.8--0.1)	3.0 (1.4-3.7)	**<0.01**
Peak TG leve (mmol/L)	21.6 (15.7-33.0)	30.3 (19.1-40.3)	19.1 (15.6-29.9)	11.2 (-8.4-25.3)	0.16
PH	7.2 (7.1-7.3)	7.3 (7.2-7.3)	7.1 (6.9-7.2)	0.2 (-0.1-0.3)	0.08
HCO3- (mmol/L)	8.2 (4.8-12.1)	12.0 (10.4-12.6)	7.0 (4.6-10.6)	5.0 (0.6-8.3)	0.08
β-hydroxybutyric acid	6.9 (4.2-9.4)	4.1 (2.3-4.7)	8.8 (6.6-10.3)	-4.7(-7.8--1.9)	**0.02**
FBG (mmol/L)	20.1 (13.5-26.2)	14.4 (12.8-15.8)	23.7 (15.8-29.3)	-9.3 (-16.0--0.9)	0.06
Cr (umol/L)	42.0 (28.5-54.6)	36.6 (31.0-60.2)	45.4 (28.3-54.1)	-8.8 (-22.7-28.9)	0.74
TC (mmol/L)	11.0 (8.0-13.5)	11.0 (9.4-14.6)	11.1 (8.0-13.4)	-0.1(-4.2-5.6)	0.79
HDL-C (mmol/L)	0.9 (0.8-1.0)	0.7 (0.7-0.9)	0.9 (0.9-1.2)	-0.2 (-0.5-0.1)	0.09
LDL-C (mmol/L)	3.7 (2.9-4.8)	4.3 (3.3-5.2)	3.2 (2.9-4.0)	1.1 (-1.0-2.5)	0.39
AST (U/L)	28.2(18.5-40.2)	35.4 (20.4-39.7)	27.8 (18.5-46.0)	7.6 (-17.5-20.7)	0.85
ALT (U/L)	18.7 (13.3-24.4)	18.4 (14.3-33.2)	19.4 (14.2-24.0)	-1.1 (-10.6-26.3)	1.00
Amylase (IU/L)	99.0 (47.0-169.2)	150.0 (94.0-201.3)	50.0 (40.5-131.5)	100.0 (-54.0-180.0)	0.28
Lipase (U/L)	137.1 (79.0-234.0)	198.6 (158.6-560.9)	84.6 (52.0-125.4)	114.0 (14.4-604.1)	**0.03**
HbA1c(%)	13.5 (12.4-15.5)	12.4 (10.9-13.3)	15.0 (13.4-15.6)	-2.6 (-4.7--0.2)	**0.02**
Antibody (n, %)	6 (28.6%)	0 (0%)	6 (42.9%)	/	0.34

DKA, diabetic ketoacidosis; SHTG, severe hypertriglyceridemia; FBG, fasting blood glucose; Cr, creatinine; TG, triglycerides; TC, total cholesterol; HDL-C, high density lipoprotein cholesterol; LDL-C, low density lipoprotein cholesterol; AST, aspartate aminotransferase; ALT, alanine aminotransferase. Variables were expressed as median and interquartile range. The Mann-Whitney U test was utilized to evaluate differences between groups. Categorical variables were summarized as frequencies and percentages [n (%)], with comparisons between groups performed using Fisher’s exact test. The bold values mean P<0.05.

In terms of treatment, upon admission, all patients were administered insulin therapy, which was continued after correction of DKA to manage hypertriglyceridemia. 7 patients with this triad were treated with plasmapheresis. As illustrated in **Table 3**, patients who received plasmapheresis had higher peak TG (median 39.3, IQR 33.0–47.1 vs. 17.0, IQR 15.6–23.6; median difference 22.3, 95% CI 9.9-37.3, *P*<0.01), higher TC (13.4, IQR 12.6–15.3 vs. 9.7, IQR 7.7–11.2; median difference 3.8, 95% CI 1.1-7.7, *P*=0.02), and higher LDL-C (5.5, IQR 4.0–5.8 vs. 3.0, IQR 2.6–3.7; median difference 2.5, 95% CI 0.4 to 3.1, *P*=0.01), but lower HDL-C levels (0.8, IQR 0.6–0.8 vs. 1.0, IQR 0.9–1.6; median difference −0.2, 95% CI −0.9 to −0.1, *P*=0.01). The median time required for TG levels to decline to 5.6 mmol/L was 12.5 days (IQR 6.0-20.0, range 1–30 days) in patients treated without plasmapheresis, compared with 3.0 days (IQR 1.0-5.0, range 1–10 days) in patients treated with plasmapheresis. However, given the small sample size and the multiple comparisons performed across **Tables 2** and **3**, all statistical analyses should be considered exploratory. *P* values are presented as descriptive measures of association and should be interpreted with caution rather than as confirmatory evidence. **Table 4** shows the changes in blood lipid and pancreatic enzyme levels before and after plasma exchange in individual patients. Nevertheless, these results should be interpreted with caution due to the potential selection bias and the retrospective nature of the study design. With the exception of three patients who were lost to follow-up, the remaining patients who underwent plasmapheresis maintained normal blood lipid levels during the follow-up period. Venous thrombotic complications were identified in four patients (57.1%) among the patients treated with plasmapheresis; however, all of the four patients recovered in the follow-up.

**Table 3:** Comparison of biochemical characteristics between DKA patients complicated by SHTG with or without plasmapheresis.

Variables	Overall (n=21)	With plasmapheresis (n=7)	Without plasmapheresis (n=14)	Median difference (95% CI)	*P*-value
Age	11.3 (10.5-13.2)	11.3 (10.7-12.9)	11.4 (8.4-13.9)	-0.1 (-2.9-4.0)	0.88
Male (n, %)	8 (38.1%)	3 (42.9%)	5 (35.7%)	/	1.00
BMI Z-score	-0.5 (-1.4-1.2)	-1.0 (-2.1-1.9)	-0.2 (-1.3-0.7)	-0.8 (-2.9-3.0)	0.97
Peak TG leve (mmol/L)	21.6 (15.7-33.0)	39.3 (33.0-47.1)	17.0 (15.6-23.6)	22.3 (9.9-37.3)	**<0.01**
PH	7.2 (7.1-7.3)	7.1 (7.0-7.2)	7.2 (7.1-7.3)	-0.1 (-0.4-0.0)	0.12
HCO3- (mmol/L)	8.2 (4.8-12.1)	6.4 (4.3-12.1)	8.9 (5.6-12.4)	-2.5(-7.4-5.5)	0.53
β-hydroxybutyric acid	6.9 (4.2-9.4)	9.2 (5.0-9.4)	6.3 (2.9-9.2)	2.9 (-3.6-6.6)	0.43
FBG (mmol/L)	20.1 (13.5-26.2)	15.9 (13.8-23.4)	20.1 (13.7-28.1)	-4.2 (-12.9-9.9)	0.85
Cr (umol/L)	42.0 (28.5-54.6)	54.2 (44.1-55.7)	36.2 (26.2-52.4)	18.0 (-5.1-28.8)	0.15
TC (mmol/L)	11.0 (8.0-13.5)	13.4 (12.6-15.3)	9.7 (7.7-11.2)	3.8 (1.1-7.7)	**0.02**
HDL-C (mmol/L)	0.9 (0.8-1.0)	0.8 (0.6-0.8)	1.0 (0.9-1.6)	-0.2 (-0.9--0.1)	**0.01**
LDL-C (mmol/L)	3.7 (2.9-4.8)	5.5 (4.0-5.8)	3.0 (2.6-3.7)	2.5 (0.4-3.1)	**0.01**
AST (U/L)	28.2(18.5-40.2)	39.2 (29.1-44.9)	21.6 (17.4-35.4)	17.6 (-5.3-31.1)	0.09
ALT (U/L)	18.7 (13.3-24.4)	18.7 (17.6-21.3)	20.4 (12.4-24.6)	-1.8 (-6.9-7.9)	0.85
Amylase (IU/L)	99.0 (47.0-169.2)	146.0 (64.0-188.5)	63.0 (40.5-150.3)	83.0 (-94.6-180.0)	0.56
Lipase (U/L)	137.1 (79.0-234.0)	140.7 (112.0-360.0)	86.2 (58.7-193.1)	54.4 (-79.2-386.5)	0.27
HbA1c(%)	13.5 (12.4-15.5)	13.3 (11.6-14.2)	14.9 (13.2-15.5)	-1.6 (-4.3-1.5)	0.26
Antibody (n, %)	6 (28.6%)	3 (42.9%)	3 (21.4%)	/	0.35

DKA, diabetic ketoacidosis; SHTG, severe hypertriglyceridemia; FBG, fasting blood glucose; Cr, creatinine; TG, triglycerides; TC, total cholesterol; HDL-C, high density lipoprotein cholesterol; LDL-C, low density lipoprotein cholesterol; AST, aspartate aminotransferase; ALT, alanine aminotransferase. The Mann-Whitney U test was utilized to evaluate differences between groups. Categorical variables were summarized as frequencies and percentages [n (%)], with comparisons between groups performed using Fisher’s exact test. The bold values mean P<0.05.

**Table 4:** Changes of blood lipids and pancreatic enzymes before and after plasma exchange in patients with DKA combined with SHTG.

Variables	No. 15	No. 16	No. 17	No. 18	No. 19	No. 20	No. 21
TG-before (mmol/L)	16.5	53.0	30.9	22.4	39.3	69.3	35.0
TG-after (mmol/L)	0.8	9.4	6.3	9.8	1.6	7.0	10.8
TC-before (mmol/L)	10.8	14.8	13.1	10.3	16.2	15.8	12.0
TC-after (mmol/L)	4.0	7.6	8.2	7.3	5.3	4.3	4.2
HDL-C-before (mmol/L)	0.8	1.0	0.6	0.6	0.8	0.6	0.8
HDL-C-after (mmol/L)	0.9	1.0	1.1	0.7	1.3	0.5	0.3
LDL-C-before (mmol/L)	4.1	6	5.5	3.3	6.6	5.6	3.1
LDL-C-after (mmol/L)	2.5	3.4	2.5	4.3	3.2	1.7	1.5
Amylase-before (IU/L)	486	81	146	31	227	150	47
Amylase-after (IU/L)	153	65	68	40	51	57	542
Lipase-before (U/L)	254.5	87	137.1	226.8	656.3	465.5	52.2
Lipase-after (U/L)	17	68	36.5	303.2	103.7	144.9	61.8

DKA, diabetic ketoacidosis; SHTG, severe hypertriglyceridemia; TG, triglycerides; TC, total cholesterol; HDL-C, high density lipoprotein cholesterol; LDL-C, low density lipoprotein cholesterol.

## Discussion

SHTG is a rare but clinically significant complication in patients with DKA. Currently, the prevalence of SHTG has been estimated to be 8% in the adult population with DKA, while it is extremely rare in pediatric patients with DKA. So far, the literature on SHTG in pediatric patients with DKA is sparse and primarily consists of case reports. In 2025, Macke et al. presented a single-center experience on SHTG, reporting on 5 pediatric patients with new-onset T1DM. Our present study reviewed the clinical management and outcomes of 21 pediatric with DKA complicated by SHTG, with a particular focus on 7 cases who underwent plasma exchange. This study provided the largest single-center study of pediatric DKA complicated by SHTG to date.

Although SHTG is a rare complication in pediatric patients with DKA, it is essential to screen for dyslipidemia in children with DM, particularly in those presenting with DKA. Richardson et al. conducted the largest retrospective study on hypertriglyceridemia by reviewing all causes of SHTG in children over a 17-year-period (1999–2016), reporting 124 cases of SHTG, with approximately 19% associated with T1DM and 15% with DKA (16). In our present study, 17 cases were newly diagnosed with diabetes, and 4 cases had a history of pre-existing diabetes. Notably, 3 of these 4 patients had self-discontinued insulin prior to this admission, further indicating that insulin deficiency plays a significant role in the pathogenesis of both DKA and SHTG.

Given insulin deficiency is considered the primary cause of SHTG in DKA, insulin is established as the first-line treatment. Even after DKA has resolved, intravenous insulin should be continued until TG levels <5.6 mmol/L (12, 22). In our present study, the TG level of case 1 was effectively reduced from 26.8 mmol/L to 1.7 mmol/L within 2 days through intravenous insulin administration. This study, along with previous pediatric case reports, demonstrates the efficacy of insulin in reducing TG levels (12, 14, 23, 24). However, a previous study by Macke et al. reported that in pediatric patients with SHTG and DKA, the average time for TG levels decline to <5.6 mmol/L was 23.8 days (16). In our present study, the median time for the level of TG to decline to 5.6 mmol/L was 12.5 days (IQR 6.0-20.0, range 1–30 days) in patients treated with insulin alone, with 6 patients requiring more than 15 days for TG levels to decrease to <5.6 mmol/L. Several factors may affect the rate of TG clearance in pediatric DKA. The increasing prevalence of obesity and insulin resistance in the pediatric population may delay TG clearance by promoting hepatic very low-density lipoprotein overproduction and impairing peripheral lipoprotein lipase activity (25). Additionally, the extremely elevated TG concentrations observed in some patients (peak TG up to 69.3 mmol/L) suggest the potential presence of underlying inherited lipid disorders, such as lipoprotein lipase (LPL) deficiency, apolipoprotein C-II (APOC2) deficiency, Apolipoprotein A5 (APOA5) variants, or Glycosylphosphatidylinositol-Anchored High-Density Lipoprotein-Binding Protein 1 (GPIHBP1) defects. Monogenic forms of hypertriglyceridemia are typically characterized by severe elevation in TG levels (>11.2 mmol/L) and may be unmasked by metabolic stressors such as DKA. Although none of our patients had a known family history of hypertriglyceridemia or consanguinity, the lack of comprehensive genetic testing constitutes a significant limitation of this study. Future prospective studies that incorporate targeted genetic sequencing for candidate genes (*LPL*, *APOC2*, *APOA5*, *GPIHBP1*, *LMF1*), are warranted to elucidate the role of monogenic disorders to DKA-associated SHTG in the pediatric population.

Notably, patients with SHTG are at risk of additional complications including acute pancreatitis. It has been reported that TG levels exceeding 11.2 mmol/L are associated with a 5% risk of developing acute pancreatitis, which could rise to 10%–20% if TG levels over 22.4 mmol/L (10). Acute pancreatitis occurs in about 26.3% (5/19) of pediatric patients with SHTG and DKA (16). Consistent with these findings, our study also identified a high incidence of acute pancreatitis in pediatric patients with SHTG and DKA (33.3%, 7/21). In our cohort, patients with pancreatitis had significantly higher BMI Z-scores compared with those without pancreatitis (median 1.8 vs. −1.2, *P*<0.01), suggesting that the underlying adiposity and insulin resistance may contribute to both the severity of hypertriglyceridemia and the risk of pancreatitis. All 7 patients with pancreatitis were aged 10 years or older, indicating that age may be a relevant risk factor, although the small sample size precludes definitive conclusions. Although the specific mechanisms underlying this association remained obscure, persistent and profound hyperlipidemia is an identifiable risk factor for acute pancreatitis (26–29). In patients who do not achieve satisfactory therapeutic outcomes with insulin, therapeutic plasma exchange may be initiated urgently for rapid reduction in TG levels (30).

In 1978, plasma exchange therapy was first used to treat severe diabetic hypertriglyceridemia (31), and later in 1982 for treating acute pancreatitis (32). Experience of plasmapheresis is largely derived from patients with hypertriglyceridemia- induced acute pancreatitis. Compared to conventional treatment, plasmapheresis has been associated with reductions in TG levels and length of hospital stay in previous studies (33–35). According to the guidelines from the French Society of Anesthesia and Intensive Care Medicine and the French National Society of Gastroenterology in 2021, initiation of therapeutic plasma exchange is recommended in patients with hypertriglyceridemia-induced acute pancreatitis who failed to respond to conventional medical therapy (36). In 2012, Lutfi et al. reported the first pediatric case presenting with DKA, SHTG, and acute pancreatitis successfully treated with plasmapheresis (13). The TG levels declined from 118.5 mmol/L to 81.3 mmol/L after 30 hours with insulin treatment, whereas the abdominal pain and poor perfusion of the patients did not improve significantly. Her abdominal pain resolved, and the TG level declined rapidly after plasmapheresis (13). To our knowledge, only 4 pediatric cases of DKA and SHTG treated with plasmapheresis have been reported in the literature, 3 of 4 cases were complicated by acute pancreatitis (13, 17, 18). In 2024, Huang et al. reported the first pediatric case of DKA and SHTG successfully treated with plasmapheresis to avoid the development of acute pancreatitis (30). In our present study, 7 patients with DKA and SHTG were treated with plasmapheresis. Notably, 4 of the 7 patients with DKA and SHTG received plasmapheresis to prevent development of acute pancreatitis. The median time for TG levels decline to 5.6 mmol/L was 12.5 days (IQR 6.0-20.0, range 1–30 days) in patients treated without plasmapheresis, compared with 3.0 days (IQR 1.0-5.0, range 1–10 days) in patients treated with plasmapheresis. It is crucial to note that patients selected for plasmapheresis differed systematically from those managed with insulin alone: they had significantly higher baseline TG, TC, and LDL-C levels. The observed faster TG decline in the plasmapheresis group may partly reflect the mechanical removal of lipids by the procedure itself, but the contribution of concurrent insulin therapy and the natural history of the disease cannot be disentangled in this retrospective design. Therefore, this is an observational finding and does not establish therapeutic superiority.

As mentioned above, there are very few available reports on the use of plasmapheresis in pediatrics with DKA and SHTG. Our observational data suggest that plasmapheresis was associated with a more rapid reduction in TG levels, although these preliminary findings do not demonstrate causality and require validation in larger, prospective studies. However, plasmapheresis is an invasive technique and requires central venous access with associated complications, such as deep venous thrombosis (37). In the present study, venous thrombotic complications were identified in four patients (57.1%) among the patients treated with plasmapheresis, although all four patients recovered during follow-up. Several mechanisms may contribute to this elevated thrombotic risk. First, DKA itself induces a prothrombotic state through hemoconcentration, endothelial activation, increased platelet aggregation, and elevated levels of procoagulant factors (38). Second, hypertriglyceridemia​may promote a procoagulant milieu through interactions between lipoproteins and the coagulation cascade (39). Third, central venous catheterization—required for plasmapheresis—is an independent risk factor for deep vein thrombosis in critically ill children, particularly in the femoral or internal jugular veins (40, 41). Previous pediatric reports on plasmapheresis for DKA-related SHTG have rarely commented on thrombotic complications, possibly due to underreporting or small sample sizes. From a clinical perspective, these findings highlight the importance of carefully assessing the risks and benefits before initiating plasmapheresis in pediatric patients with DKA. Future studies are needed to determine the efficacy of plasmapheresis in the treatment of pediatric patients with DKA and SHTG.

There are some limitations in this study. Firstly, owing to the retrospective nature and the small sample size of the study, no conclusions regarding causality or treatment superiority can be made from these data. Secondly, the small sample size limited statistical precision and prevented robust adjustment for potential confounding factors. Thirdly, as a single-center study, the conclusions cannot be generalized to the entire Chinese population. Fourthly, the absence of genetic testing for inherited lipid disorders limits our ability to distinguish monogenic forms of hypertriglyceridemia from acquired DKA-induced SHTG. Therefore, prospective, multicenter studies with standardized protocols, genetic testing, and systematic adverse event monitoring are needed to address these limitations.

In conclusion, our study underscores the importance of routine lipid screening in pediatric patients presenting with DKA. Acute pancreatitis is an important complication of DKA associated with SHTG, particularly in children aged 10 years or older. Insulin therapy remains the cornerstone of management for pediatric DKA with SHTG. However, in children who show unsatisfactory reduction in TG levels or persistent abdominal pain despite insulin treatment, prompt initiation of plasma exchange may be considered. The optimal timing, benefits, and risks of plasma exchange in this population warrant further investigation.

## Data Availability

The original contributions presented in the study are included in the article/**Supplementary Material**. Further inquiries can be directed to the corresponding author.
