# Supplementary material for: Insulin is central, plasmapheresis is salvage: clinical spectrum and management of severe hypertriglyceridemia in 21 children with diabetic ketoacidosis
Source: Front Endocrinol (Lausanne). 2026 Aug 26;17:1920206. doi: 10.3389/fendo.2026.1920206 (PMC13553409; doi:10.3389/fendo.2026.1920206)
Supplement: Supplementary file 1 [file Table1.docx]

Supplementary Table 1. Clinical characteristics of patients with DKA complicated by SHTG

| No | Age | Gender | BMI  (kg/m^2^） | BMI Z-score | Type of diabetes | New-onset diabetes | Peak TG  level (mmol/L） | Cerebral  edema | Acute  pancreatitis | Plasmapheresis | Thrombosis | lipid-lowering agent | Time to normalized TG level (days, (TG level, mmol/L) | Follow-up  (TG level, time) |
| --- | --- | --- | --- | --- | --- | --- | --- | --- | --- | --- | --- | --- | --- | --- |
| 1 | 5y | M | 15.7 | 0.2 | T1DM | Yes | 26.8 | No | No | No | No | No | 2d (1.7) | 0.3 (1 month) |
| 2 | 14.3y | M | 31.8 | 2.1 | T2DM | Yes | 15.7 | No | Yes | No | No | No | 16d (2.3) | 5.8 (6 momths) |
| 3 | 10.8y | F | 21.5 | 1.2 | DM | Yes | 30.3 | No | Yes | No | No | No | 17d (3.2) | NA |
| 4 | 12.9y | M | 17.3 | -0.5 | DM | Yes | 15.7 | Yes | No | No | No | No | 2d (2.1) | 6.9 (3 years) |
| 5 | 14.2y | F | 17.5 | -0.8 | DM | Yes | 20.3 | No | Yes | No | No | Yes | 21d (6.9) | NA |
| 6 | 11.1y | F | 13.9 | -2.1 | T1DM | Yes | 21.6 | No | No | No | No | No | 14d (1.59) | 1.44 (3 months) |
| 7 | 12.4y | F | 21.4 | 0.9 | T2DM | Yes | 17.8 | No | Yes | No | No | No | 25d (1.89) | 4.77 (2 months) |
| 8 | 7.6y | F | 13.2 | -1.9 | T1DM | Yes | 13.3 | No | No | No | No | No | NA | 0.6 (1 month) |
| 9 | 15.8y | F | 20.3 | 0 | DM | No | 24.2 | No | No | No | No | No | 5d (3.5) | NA |
| 10 | 15.9y | F | NA | NA | DM | No | 33.0 | No | No | No | No | No | 12d (3.7) | NA |
| 11 | 11.7y | M | 15.2 | -1.4 | T1DM | Yes | 11.3 | No | No | No | No | No | 15d (1.3) | 0.8 (3 months) |
| 12 | 2.0y | M | 16.5 | 0 | T1DM | Yes | 15.6 | No | No | No | No | No | 3d (1.5) | 1.0 (2 months) |
| 13 | 11.1y | F | 23.0 | 1.4 | T1DM | Yes | 11.1 | No | No | No | No | No | 1d (3.2) | 0.5 (3 months) |
| 14 | 6y | F | 13.9 | -1.1 | T1DM | Yes | 16.2 | No | No | No | No | No | 30d (0.6) | 0.6 (6 months) |
| 15 | 11.3y | F | 12.5 | -3.4 | T1DM | No | 16.5 | Yes | No | Yes | Yes | No | 1d (0.8) | 0.8 (3 momths) |
| 16 | 13.2y | F | 16.4 | -1.0 | T1DM | Yes | 53.0 | No | No | Yes | Yes | No | 2d (2.4) | 0.6 (3 momths) |
| 17 | 6.5y | F | 12.5 | -2.8 | T1DM | Yes | 30.9 | Yes | No | Yes | No | No | 3d (1.1) | 0.6 (6 momths) |
| 18 | 10.8y | M | 25.6 | 2 | T2DM | Yes | 41.3 | Yes | Yes | Yes | Yes | No | 10d (8.8) | NA |
| 19 | 10.5y | M | 35.2 | 2.6 | T2DM | Yes | 39.3 | No | Yes | Yes | No | No | 2d (1.6) | NA |
| 20 | 17.4y | M | 29.3 | 1.8 | T2DM | No | 69.3 | No | Yes | Yes | Yes | No | 5d (2.91) | NA |
| 21 | 12.6y | F | 15.4 | -1.4 | DM | Yes | 35.0 | Yes | No | Yes | No | No | 2d (3.5) | 1.5 (1 month) |

Abbreviations: DKA, diabetic ketoacidosis; SHTG, severe hypertriglyceridemia; PH, body mass index; TG, triglycerides; M, male; F, female; DM, diabetes mellitus; T1DM, type 1 diabetes mellitus; T2DM, type 2 diabetes mellitus; NA, not available.
